# Exploring sociodemographic subgroup differences in multiple mini-interview (MMI) performance based on MMI station type and the implications for the predictive fairness of the Hamburg MMI

**DOI:** 10.1186/s12909-019-1674-z

**Published:** 2019-07-03

**Authors:** Mirjana Knorr, Hubertus Meyer, Susanne Sehner, Wolfgang Hampe, Stefan Zimmermann

**Affiliations:** 1 Institute of Biochemistry and Molecular Cell Biology, University Medical Center Hamburg-Eppendorf (UKE), N30, Martinistraße 52, 20246 Hamburg, Germany; 2 Institute of Medical Biometry and Epidemiology, University Medical Center Hamburg-Eppendorf (UKE), W34, Martinistraße 52, 20246 Hamburg, Germany

**Keywords:** Multiple mini-interview, Sociodemographic subgroup differences, Predictive fairness, Gender differences, Gender and age interaction, Native language, Medical family background

## Abstract

**Background:**

Sociodemographic subgroup differences in multiple mini-interview (MMI) performance have been extensively studied within the MMI research literature, but heterogeneous findings demand a closer look at how specific aspects of MMI design (such as station type) affect these differences. So far, it has not been investigated whether sociodemographic subgroup differences imply that an MMI is *biased*, particularly in terms of its predictive validity.

**Methods:**

Between 2010 and 2017, the University Medical Centre Hamburg-Eppendorf (UKE) tested 1438 candidates in an MMI who also provided sociodemographic data and agreed to participate in this study. Out of these, 400 candidates were admitted and underwent a first objective structured clinical examination (OSCE) after one and a half years, including one station assessing communication skills. First, we analyzed the relationship between gender, age, native language and medical family background and MMI station performance including interaction terms with MMI station type (simulation, interview, and group) in a hierarchical linear model. Second, we tested whether the prediction of OSCE overall and communication station performance in particular differed depending on sociodemographic background by adding interaction terms between MMI performance and gender, age and medical family background in a linear regression model.

**Results:**

Young female candidates performed better than young male candidates both at interview and simulation stations. The gender difference was smaller (simulation) or non-significant (interview) in older candidates. There were no gender or age effects in MMI group station performance. All effects were very small, with the overall model explaining only 0.6% of the variance. MMI performance was not related to OSCE overall performance but significantly predicted OSCE communication station performance with no differences in the prediction for sociodemographic subgroups.

**Conclusions:**

The Hamburg MMI is fair in its prediction of OSCE communication scores. Differences in MMI station performance for gender and age and their interaction with MMI station type can be related to the dimensions assessed at different station types and thus support the validity of the MMI. Rather than being threats to fairness, these differences could be useful for decisions relating to the design and use of an MMI.

## Background

### Introduction

Admission into medical school is a demanding competition between highly competent candidates. It is in the interests of both medical schools and candidates that the selection tools used during the admission process are reliable, valid and fair [1].

Criticism of the reliability of traditional interviews has led to the rise of multiple mini-interviews (MMIs) to assess non-academic skills at medical schools around the globe [2]. An MMI is a highly structured procedure which consists of a series of short (usually 5–10 min) interviews [3]. Candidates rotate from one interview room to the next and are assessed by one or two independent raters. The method aims to reduce interviewer bias and increase reliability [4]. MMIs are flexible and can be adjusted to the needs and aims of individual institutions [2]. So far, the accumulated research evidence suggests that MMIs are more reliable and valid than traditional interviews [5].

Despite many studies investigating the relationship between sociodemographic factors and performance at MMIs, the picture is still unclear. Results are ambiguous, with some institutions reporting significant differences in MMI performance based on gender, age or other variables, while other studies have found no such differences [6]. In our summary of the latest MMI research, we concluded that these heterogeneous findings most likely stem from the different MMI designs and should be further explored [7]. Likewise, in their recent systematic review of MMIs for undergraduate student selection, Rees et al. [3] recommended further exploration of the performance of minority groups and possible bias in MMIs. These suggestions can be summarized into two research questions:

1. How do specific aspects of MMI design relate to performance differences between sociodemographic subgroups?
2. Do sociodemographic subgroup differences in MMI performance indicate that an MMI is biased or unfair?

The first question aims to better understand the underlying mechanisms that lead to differences between sociodemographic subgroups. Stations with unexpected subgroup differences might put their fairness into question and thereby jeopardize their re-use. This would also have negative effects on the cost-effectiveness of an MMI as the development of an MMI station is very costly [8]. Therefore, station developers and institutions thinking about implementing an MMI would benefit from increased knowledge about the interplay between measurement intention, station design and what subgroup differences may be expected.

For the second research question, it is important to first clarify the concept of test fairness. A fair test “reflects the same construct(s) for all test takers, and scores from it have the same meaning for all individuals in the intended population” [[9], p., 50]. The fairness of a test is therefore an important aspect of its validity [9]. Thus, a test should maximize construct variance and reduce construct-irrelevant variance favouring specific subgroups as far as possible [9, 10]. However, the notion that there should be no difference in performance between relevant sociodemographic groups is rather naïve, and a lack of such differences does not automatically imply that a test is fair [11, 12]. Indeed, such differences might even support the validity of a test if the varying characteristic is related to the construct of interest. One suggestion to analyse test fairness has been to investigate whether the relationship between a test score and a relevant outcome is the same for different sociodemographic groups (“predictive fairness”) [9, 12]. If the predictive value of an MMI depends on sociodemographic background, the results may confound its intended use and thus limit its validity. Despite its importance, the predictive fairness of MMIs has garnered limited attention this far.

In this multi-cohort study of the Hamburg MMI, we want to address both research questions by

1. exploring how the relationship between sociodemographic factors and MMI station performance differs depending on MMI station type
2. analysing the predictive fairness of the Hamburg MMI.

### The Hamburg MMI

The University Medical Center Hamburg-Eppendorf (UKE) piloted the first German MMI for admission into medical school in 2009 [8]. Since then, the MMI has become an integral part of the yearly selection process. International candidates from other European Union (EU) countries are required to go through the same selection process as German candidates. The Hamburg MMI was designed to assess psychosocial competencies and predominantly focuses on simulation scenarios and interview questions that require interpersonal skills (i.e., empathy and communication skills) and self-regulation, with a strong focus on self-reflection. As of 2016, teamwork skills have been added to the list of dimensions and are assessed via group stations. Teamwork skills are defined as a combination of leadership, collective problem solving and team orientation. In contrast, ethical reasoning and clinical knowledge and skills are excluded from the psychosocial skills construct. Previous research into the Hamburg MMI has supported its reliability [8] and demonstrated initial evidence of its predictive validity [13].

Given the measurement intention and the design of the MMI, we decided to concentrate on the relationship between MMI performance and four relevant sociodemographic factors, namely gender, age, language and medical family background. To address our research aims, we considered

1. station type as a discrete feature of MMI design
2. OSCE performance as a criterion for predictive fairness.

Station type is one of the most basic and controllable aspect of MMI design, with considerable variance between institutions, ranging from interview-station-only MMIs [14] to combinations of interview and roleplay stations [4] and the further addition of collaborative and group stations [15, 16].

We chose OSCE results as the criterion for predictive fairness because they have proven to be the most consistent criterion for analysing the predictive validity of MMIs [7] and we were previously able to demonstrate this relationship for the Hamburg MMI in particular [13].

### Assumptions

#### Gender and age

Previous analyses of gender and age differences in MMI performance were either non-significant or mostly pointed in the same direction, with female [17, 18] and older [19, 20] candidates achieving higher MMI scores. The same tendency was also found for the 2014 cohort of the Hamburg MMI [13].

Two possible explanations for these gender differences have been discussed: women may truly have a higher level of the competencies of interest or they may be rated higher due to an expectancy bias [17, 20]. These gender differences would only be unfair in the latter case. For the Hamburg MMI, gender differences are expected for some of the dimensions that the stations aim to assess. Women typically score higher on measures of empathy and research into behavioural science and neuroscience suggests this could have evolutionary causes [21]. The differences seem to be especially pronounced for the affective component of empathy (i.e., sharing and responding to emotions) as compared to the cognitive component (i.e., perspective taking) [22]. For self-reflection, there are indications that women more strongly engage in self-reflection and are more self-conscious [23]. In contrast, there seem to be no general gender differences for leadership effectiveness [24] or engagement in collaboration [25]. At simulation stations, candidates directly interact with a simulated patient and respond to the emotions displayed by the actor (i.e., affective empathy). Interview stations, where candidates discuss hypothetical scenarios, instead tap into cognitive processes such as perspective taking (i.e., cognitive empathy) and self-reflection. Finally, group stations aim to measure teamwork skills which are conceptualized as a combination of leadership and collaboration. On this basis, we assume that female candidates should perform significantly better than male candidates would at simulation stations and interview stations but not at group stations **(Assumption 1A)**.

Age differences are typically explained by the maturity and life experience of older candidates [20, 26] which help them develop higher levels of competency. Maturity effects can also moderate gender differences. Girls seem to mature earlier than boys in terms of both physical [27] and neurological development [28] and also in increased perspective taking and prosocial behaviour [29, 30]. Given that many candidates for medical school in Germany are direct school-leavers around the age of 18, it is possible that the interpersonal skills of young male candidates will catch up to those of their female counterparts. If the maturity hypothesis holds true, age should moderate the relationship between gender and station performance **(Assumption 1B)**.

#### Language

The intended use of the Hamburg MMI is to select from a pool of candidates that includes immigrants with German citizenship and EU foreigners, some of whom do not speak German as their native language. However, major language barriers compared with their native language might make it difficult for candidates to demonstrate their true level of communication skills and could interfere with raters’ assessment. Indeed, a study from Ireland found that non-native speakers exhibited weaker performance [31], while a study from Canada found the same effect only in female candidates [20].

Direct interaction with simulated patients could provide a chance for candidates to express empathy non-verbally and use simpler language, whereas interview stations rely more heavily on verbal communication and often require a formal academic vocabulary register. At group stations, non-native speakers might find it difficult to follow the discussion and to contribute their own thoughts if the other group members are native speakers. We therefore expect that non-native German speakers will perform worse at all station types, with a less pronounced effect at simulation stations **(Assumption 1C)**.

#### Medical family background

The Hamburg MMI intends to measure psychosocial competencies that are independent of prior medical knowledge, but some scenarios at simulation, interview and group stations are set in a medical context. Some candidates taking the MMI have already gained medical knowledge and skills, either directly because of their previous education and work experience in the healthcare system or indirectly because close family members are physicians and have studied medicine themselves. In the latter case, these family members might have transferred certain knowledge and skills for handling typical critical situations in a medical context. More broadly, they might have shaped general values and attitudes in the family via their own personal development working in this profession. Growing up in a medical family background could therefore increase the chances that a candidate is already familiar with MMI-relevant topics and situations, which might lead to an advantage.

Candidates with a medical family background seem especially attracted to medical studies [32]. In Germany, a significantly higher percentage of medical graduates has at least one parent working as a physician compared to other disciplines [33]. Simmenroth-Nayda and Görlich [34] found that candidates who had physicians in their family did not perform differently at the MMI for admission to the University of Göttingen. However, the category “family member” was not limited to close family members (e.g., parents) but also included distant family members (e.g., uncles/aunts, grandparents, etc.). We argue that transfer of MMI-relevant knowledge, skills, values and attitudes is more likely from close family members who live in the same household. Therefore, we expect that candidates whose parents are physicians will perform better for each station type **(Assumption 1D).**

#### Predictive fairness

According to the definition of predictive fairness as described above, the relationship between test scores and relevant outcomes should be the same for different sociodemographic subgroups. Consequently, there should be no differences in an MMI’s prediction of OSCE results related to gender, age, language or medical family background **(Assumption 2).**

## Methods

### Data collection and sampling

#### Admission process

In accordance with current German admission regulations, 40% of medical students are selected through national quotas that rely on GPA or waiting time while the other 60% are selected based on university-specific selection criteria. Since 2010, the University Medical Center Hamburg-Eppendorf (UKE) has conducted its admissions process in two steps. Every year, candidates are invited to the HAM-Nat, a multiple-choice natural sciences test [35]. Based on a combination of HAM-Nat score and Abitur grade (the German secondary school leaving grade), the top 115 candidates (approximately) are admitted directly. In the second step, the 200 candidates with the next highest performance are invited to the MMI. After the MMI, around 100 candidates will be admitted based on a combination of Abitur grade, HAM-Nat score and MMI score. During the admission process, candidates are invited to complete a voluntary online questionnaire providing information on demographic data, previous education, language and family background.

#### MMI sample

For the initial analysis of the relationship between sociodemographic factors and MMI performance, we included all candidates who participated in the MMI between 2010 and 2017 and who also completed the sociodemographic questionnaire and gave their written informed consent. Candidates who are not admitted have the chance to apply again, with no limit to the number of applications. While two studies found a tendency for repeat MMI participation to lead to improved performance [36, 37], the impact of previous MMI participation on later attempts has not been fully explored. Therefore, we decided to only analyse MMI performance at the first attempt.

#### Medical studies

In 2012, the UKE introduced its new curriculum for medical school (“integrierter Modellstudiengang Medizin (iMed)”). Unlike the previous curriculum, where objective structured clinical examinations (OSCEs) were rare, the new curriculum has several obligatory OSCEs. The first is to be taken after one and a half years.

#### Student sample

At the time of the data analysis, students who were admitted in 2015 were two years into their medical studies and most had taken the first OSCE of the curriculum. Thus, for the analysis of predictive fairness, we included all medical students who gave their informed consent, provided sociodemographic data and participated in the MMI between 2012 and 2015. Students admitted in 2010 and 2011 were excluded because they were part of the old curriculum that did not provide comparable OSCE results.

### Study measures

#### Multiple mini-interview

Between 2010 and 2017, each MMI consisted of at least six simulation and interview stations. From 2016, two group stations were added (Table 1). Each individual station lasted five minutes and overall candidate performance for the interaction with the interviewer or actor was rated by two independent raters on a Likert scale ranging from 1 (very poor) to 5 (very good). Rater pairs were usually balanced in terms of gender (one male, one female) and profession (one psychologist, one physician). Group stations were typically 12 to 15 min long and rated by two independent raters on two five-point Likert scales for the domains “leadership and problem solving” and “team orientation”. Each team consisted of three candidates, and team composition was changed between the two group stations. Except for 2013, the MMI yielded an overall reliability of G > 0.60 (see Hissbach et al. [8] for more details on the calculation). Three variables were used for the data analysis:

- *MMI station performance:* A candidate’s station performance was the mean of the available ratings within an individual station. If only one rater was present, then this rating was taken as station performance.
- *Station type:* Categorical variable with 1 = simulation station, 2 = interview station, and 3 = group station.
- *zMMI overall*: The overall MMI score was calculated as the mean of all station scores. As the content and number of stations varied between MMIs each year, the overall MMI scores for candidates within each year were *z*-standardized to make scores more comparable between the years. This approach had been used in comparable settings where *z*-standardization was used to compare MMI values between different schools [18, 26].

**Table 1:** MMI characteristics for each year, study samples and frequencies for sociodemographic variables within each sample

Cohort	No. of interview / simulation / group stations	Overall reliability^1^	Overall No. of MMI participants	MMI sample^2^ (No.)	Student sample^3^ (No.)
2010	4 / 5 / 0	.76	193	180	0
2011	3 / 5 / 0	.68	194	184	0
2012	6 / 3 / 0	.68	192	179	102
2013	3 / 3 / 0	.48	198	187	111
2014	4 / 3 / 0	.65	194	179	95
2015	5 / 3 / 0	.62	192	178	92
2016	4 / 3 / 2	.67	190	171	0
2017	4 / 3 / 2	.68	198	180	0
Total	33 / 28 / 4			*N* = 1438	*N =* 400
Sociodemographic variables					
Male				40.8%	37.8%
Age 21 or older				34.6%	34.0%
German as first language				88.5%	89.2%
Medical family background				27.8%	30.0%

N = total sample size, n = sub-sample size, No. = number of

^1^The model for the estimation of the overall reliability was described in more detail by Hissbach et al. (2014)

^2^Candidates who participated in the study and had their first attempt at the MMI in the indicated year

^3^Medical students who had their first MMI attempt in the indicated year, participated in the study and had OSCE results. Students admitted in 2010 and 2011 were excluded because they had a different curriculum, students admitted in 2016 and 2017 did not have OSCE results at the time of data analysis

#### Sociodemographic variables

Information on gender, age, language and medical family background was taken from the sociodemographic questionnaire. In line with the analysis of the HAM-Nat by Meyer et al. [38], we used the following dichotomized variables, in which the label of the variable equals 1 and the other category equals 0:

- *Male*
- *Age 21 or older*
- *German as first language*
- *Medical family background* (i.e., at least one parent is a physician)

We set the cut-off value for the age variable to 21 in order to be able to compare our results to those from Meyer et al. [38] and the UKCAT-12 study [39]. It also seemed sensible to introduce a cut-off at 21, because older candidates most likely apply with knowledge and experiences that exceed secondary schooling.

#### OSCE performance

The first OSCE, taken after one and a half years of the curriculum, consists of 12 stations and measures basic clinical skills. One of the stations aims to assess communication skills. Although the content of the stations has remained the same over the years, the rating criteria and the maximum score for each station changed from 10 in earlier years to 20 since 2016. The following two variables were considered as outcome criteria in the analyses:

*OSCE overall performance:* Percentage of scores achieved over all 12 stations (i.e., 100 if a student achieved the maximum score at all stations).

*OSCE communication station:* Percentage of score achieved in the communication station (i.e., 100 if a student achieved the maximum score at this station).

### Data analysis

Sample characteristics are given as absolute and relative frequencies or mean +/− standard deviation, whichever is appropriate.

We began by analysing the relationship between sociodemographic variables and MMI performance within the MMI sample. Due to the dependent data structure, a three-level hierarchical model was employed, where stations were nested within candidates, which were nested within year. The stations were modelled as fixed effects, because we were interested in the station effect as well as the interactions with station type. Candidates and years were modelled as nested random effects and the variance structure was set to identity due to computational restrictions.

Sociodemographic variables (male gender, age 21 or older, German as first language and medical family background) were included as potential predictors. In addition, the interaction between age and gender was modelled to analyse whether possible gender effects were age dependent. Furthermore, we modelled all interaction terms between these predictors (including the age by gender interaction) with station type. This allowed us to investigate possible performance differences for these predictors with respect to station type. Variable selection was performed using a backwards elimination strategy via the likelihood ratio test.

For the resulting model, the adjusted effects with corresponding 95% confidence interval (CI) and effects size were reported. The adjusted results were also estimated as marginal means with 95% CI, which are represented in graphs. Post hoc tests to compare the estimated means were calculated with contrast tests, using Wald tests. All the described analyses were carried out in Stata/SE 15.1.

In a second step, we analysed the relationship between zMMI overall with OSCE overall performance and OSCE communication station performance to determine whether zMMI overall significantly predicted these outcomes. Next, we analysed the predictive fairness in two regression models. The first model included all main effects for MMI performance and sociodemographic variables. The second model also included interaction terms between MMI performance and sociodemographic variables in order to test whether the relationship between MMI and OSCE performance differed between sociodemographic groups. The second part of the analysis was conducted in IBM SPSS Version 21.0.0.0.

All of the models present available case analyses. A two-tailed *p* <  0.05 was considered statistically significant. Due to the explorative nature of the analyses, nominal *p*-values are reported without correction for multiplicity.

## Results

### Relationship between sociodemographic factors and MMI performance (MMI sample)

After restricting the sample to first attempt MMI candidates and taking into account candidates who did not give their informed consent, we analysed more than 90% of the MMI participants each year (e.g., 180 of 198 MMI participants in 2017 = 91%; Table 1). A majority of the MMI sample (*N* = 1438) were female (59.2%) and under the age of 21 (65.4%). The category “21 and older” mostly comprised candidates between the age of 21 and 25 (95.6%). Most candidates spoke German as their first language (88.5%), while more than a quarter had a medical family background (27.8%). Mean (3.37) and skewness (− 0.32) over all individual MMI station performances (*N* = 11,658 observations) indicated that station performances were above average (Table 2).

**Table 2:** Descriptive statistics for all continuous study variables within the two analysed samples

	*N*	*M*	*SD*	*Min*	*Max*	*Skew*	*Kurt*
MMI sample							
MMI station performance	11,658^1^	3.37	0.96	1	5	−0.32	− 0.40
Student sample							
zMMI overall	400	0.40	0.87	−2,34	2.59	−0.22	0.10
OSCE overall performance	400	85.43	4.79	68.33	97.50	−0.43	0.41
OSCE communication station	400	80.00	10.54	30.00	100.00	−0.65	1.82

*N* = sample size, M = mean, SD = standard deviation, Min = minimum value, Max = maximum value, Skew = Skewness, Kurt = Kurtosis

MMI = multiple mini-interview, zMMI = z-standardized MMI values, OSCE = objective structured clinical examination

^1^Based on 1438 first attempt candidates; three individual station performances were not available

**Fig. 1:**
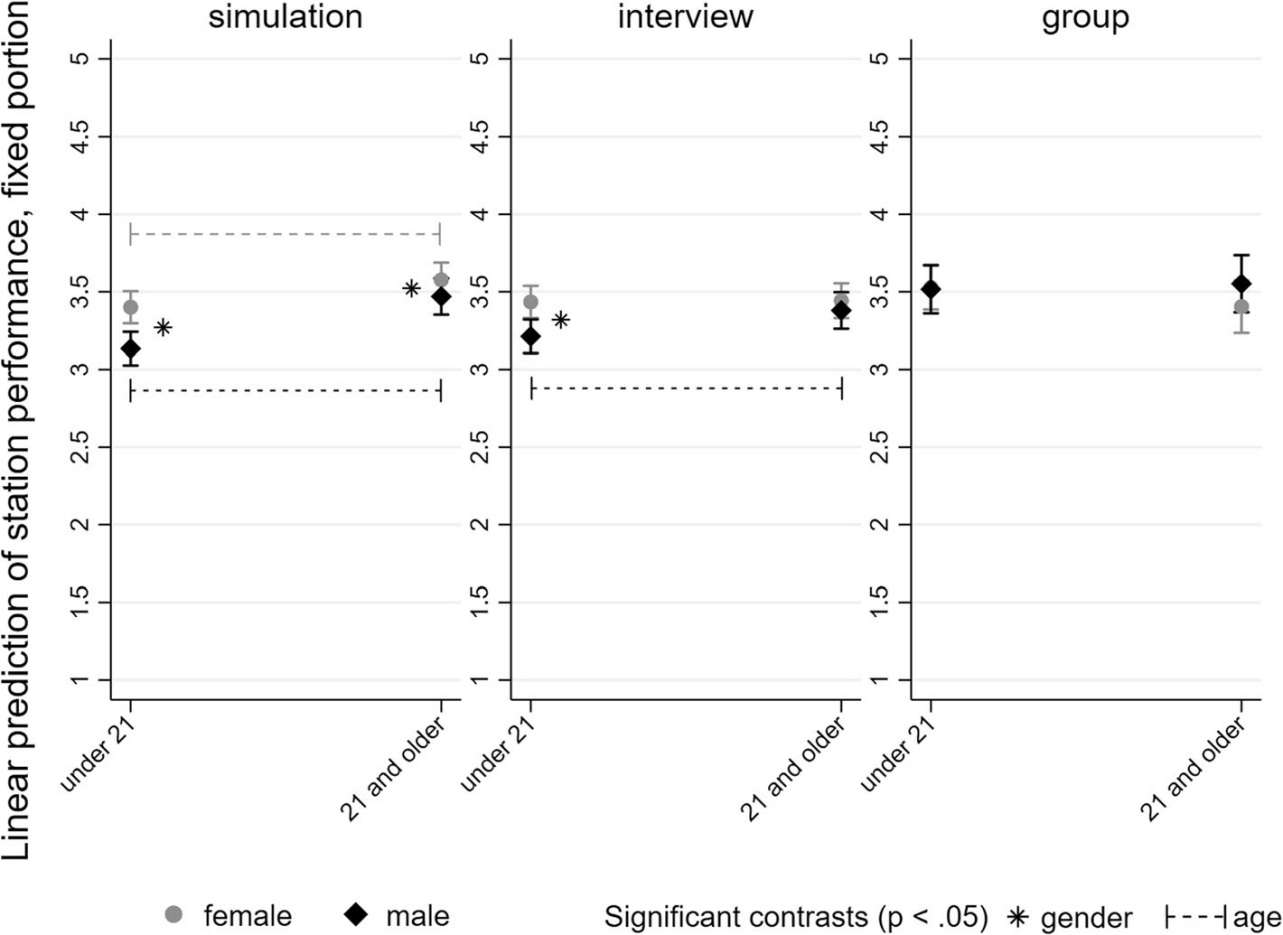
Margin plot of the interaction between gender and age displayed separately for each station type

Table 3 displays the final model resulting from the backwards elimination strategy. At only 0.6%, the percentage of the variance explained by the overall model was very low. Gender and age had a significant interaction and both variables showed interactions with station type. Figure 1 highlights significant contrasts, demonstrating that the gender gap was more pronounced for younger candidates than for older candidates in interview and simulation stations. Moreover, older candidates performed better than young candidates for both genders in simulation stations (21 and older vs under 21 for female: 0.18, 95% CI: 0.10; 0.25, *p* <  0.001; male: 0.34, 95% CI: 0.25; 0.42, *p* <  0.001). For interview stations, only male candidates showed a significant effect (0.17, 95% CI: 0.08; 0.25, *p* <  0.001). Neither gender nor age differences were detected in group stations.

**Table 3:** Hierarchical linear model predicting MMI station performance (*N* = 11,658 ratings within 1438 candidates over 8 years)

Fixed effects	*b*	*95%-CI*	*p*	*Effect size*
First language German	0.16	0.08; 0.24	< 0.001	− 0.000
Interaction terms (male vs female):				
gender x station type			0.003	0.001
gender x age			0.003	− 0.000
age x station type			< 0.001	0.002
Younger than 21				
Simulation	− 0.27	− 0.34; − 0.20	< 0.001	
Interview	− 0.22	− 0.29; − 0.15	< 0.001	
Group	− 0.01	− 0.16; 0.14	0.883	
21 and older				
Simulation	− 0.11	− 0.20; − 0.02	0.020	
Interview	− 0.06	− 0.15; 0.03	0.174	
Group	0.15	− 0.01; 0.31	0.072	
Interaction medical family background x station type (at least one vs. no parent is physician)			0.004	0.001
Simulation	− 0.06	− 0.12; 0.01	0.087	
Interview	0.07	− 0.00; 0.13	0.053	
Group	− 0.05	− 0.21; 0.11	0.572	
R^2^	.006			
Between year variance	0.02	2.0% of total variance		
Between candidate variance (within year)	0.12	15.4% of total variance		
Within rating / unexplained variance	0.77	82.6% of total variance

Candidates who spoke German as their first language performed better (*b* = 0.16, *p* <  0.001), irrespective of station type, while the effect of medical family background on MMI performance depended on station type (*p* = 0.004). However, none of the subgroup comparisons were significant.

### Predictive fairness (student sample)

Of the admitted students, 37.8% were male, 34.0% aged 21 or older, 89.2% native German speakers and 30.0% had a medical family background (Table 1). As only those MMI participants with a medium to very good MMI performance were admitted, the mean standardized MMI performance (*zMMI overall*) within the student sample is 0.40, with a standard deviation of 0.87. These students scored a mean of 85.4% in *OSCE overall performance* and 80.0% in *OSCE communication station performance,* respectively. Both OSCE measures were negatively skewed (Table 2).

MMI performance (*zMMI overall*) demonstrated a weak but significant positive correlation with performance at the *OSCE communication station* (*r* = 0.17, *p* = 0.001). However, the correlation between *zMMI overall* and *OSCE overall performance* was non-significant (*r* = 0.06, *p* = 0.20). Therefore, we concentrated on *OSCE communication station* performance as the criterion for analysing predictive fairness.

In the subsequent data analysis, the prediction model showed low tolerance values of < 0.10 for *zMMI overall* and the interaction term between *zMMI overall* and *German as first language*, which indicates problems with multicollinearity. Therefore, *German as first language* had to be excluded from the model. The first model for the prediction of OSCE communication station performance showed a significant main effect of MMI performance (*b* = 2.15, *p* = 0.001). The main effects for sociodemographic variables were all non-significant. Finally, the second model revealed no additional interaction effects (Table 4).

**Table 4:** Linear regression model predicting OSCE communication station performance (*N* = 400)

	Model 1			Model 2		
Predictors^1^	*b*	*95%-CI*	*p*	*b*	*95%-CI*	*p*
zMMI overall	2.15	0.94; 3.36	0.001	2.98	1.00; 4.96	0.003
Male	−0.004	−2.15; 2.14	0.997	0.84	−1.49; 3.16	0.481
21 and older	−0.59	−2.78; 1.60	0.597	0.03	−2.48; 2.53	0.984
At least one parent is a physician	−1.70	−3.94; 0.55	0.138	−2.36	−4.89; 0.16	0.067
Interactions						
zMMI overall * Male				−1.91	−4.35; 0.54	0.127
zMMI overall * 21 and older				−1.10	−3.65; 1.45	0.396
zMMI overall * At least one parent is a physician				1.08	−1.48; 3.64	0.409
ΔR^2^	.035			.044		

^1^As only 43 out of 400 students did not speak German as their first language, the product term of *zMMI overall* and *German as first language* was too redundant to *zMMI overall* resulting in multicollinearity. Therefore, *German as first language* had to be excluded from the model

## Discussion

This is the first large-scale analysis of sociodemographic subgroup differences in MMI performance at a German medical school. To the best of our knowledge, it is also the first study to take a closer look at possible interactions with station type and at the predictive fairness of an MMI.

Based on our first research question, we analysed whether there were differences in MMI performance depending on gender, age, first language, or medical family background and if these differences were the same for three station types. Overall, with only 0.6% of the variance in MMI station performance explained, we consider the differences we found to be marginal. We analysed a large sample, which typically yields statistically significant differences even for small effects [40]. For example, the difference of *b* = − 0.27 between young men and women in simulation stations is less than a third of the standard deviation of MMI station performance (*SD* = 0.97). Although such a difference might still be meaningful to rejected candidates with scores close to those of accepted candidates, our results do not indicate any grave imbalances that would call the fairness of the Hamburg MMI into question. Moreover, the interactions between gender, age and station types relate well to the dimensions that we aim to assess at these different station types, which could even be interpreted as narrow evidence for validity.

Our finding that subgroup differences did not substantially explain variance in MMI station performance is not a sufficient indicator for the fairness of our MMI. Therefore, we analysed the predictive fairness of our MMI based on our second research question. The analysis of candidates admitted to medical school showed that the MMI performance over four cohorts significantly predicted OSCE communication station performance and that this predictive effect did not vary depending on sociodemographic factors. These results more strongly support the fairness of the Hamburg MMI. They also strengthen the validity argument of our MMI. Based on four cohorts, we were able to demonstrate that the MMI specifically predicts an OSCE station that measures competencies which the MMI aims to assess. We did not detect this relationship in a previous study based on the 2014 MMI cohort [13], probably due to the smaller sample size and range restriction. The larger dataset in this current study also included some cases of students who were rejected at their first MMI attempt but were admitted in the following years, which might have lessened the effect of range restriction. On the other hand, we did find a positive relationship between MMI and OSCE overall performance in the previous study [7], which was not replicated in this study. Future studies focusing on the validity of the Hamburg MMI should therefore take possible cohort effects into account. Additionally, future research could also analyse whether different station types – with regard to the different dimensions these stations aim to assess – have different predictive effects on relevant outcomes. This analysis was beyond the scope of this work, which instead focused on sociodemographic factors. Furthermore, OSCE data was not yet available for MMI cohorts experiencing group stations.

Despite small effect sizes, our findings are useful for a better understanding of gender, age, language and medical family background effects in an MMI. We will therefore discuss each finding in further detail.

### Gender and age

Our study replicates findings by some other institutions that female candidates performed better [17, 18, 41]. In line with some other authors, we also found that older candidates had higher performance ratings than candidates under the age of 21 [17, 18, 26, 41]. Our results support our first assumption (1A) that gender differences would be present in simulation and interview but not in group stations. They further indicate that the gender gap in MMI performance can partly be explained by differences in maturity, thus supporting our second assumption (1B). At simulation stations, both male and female candidates perform better when they are 21 or over. However, men seem to catch up, as the difference between 21 and older males vs. females is smaller than for younger candidates. The observation is similar at interview stations, where female candidates have the same performance level in both age groups while male candidates again seem to catch up, resulting in non-significant gender differences in older age.

Although our results allow no definite conclusion that simulation stations assess affective empathy and interview stations measure perspective taking and self-reflection, they suggest that different station types tap different constructs. For a further investigation of the assumption that gender differences depend on the empathy component, we suggest that stations be classified according to the degree in which they aim to assess affective and cognitive empathy. This could allow us to test within station types whether the female advantage is especially strong at stations that more strongly require affective empathy.

Our sample included very few candidates above the age of 25. To assess the maturity hypothesis, studies on MMIs for selection into specialty training could provide more insight on whether gender differences fully disappear in older candidates. We currently know of only one study about an MMI used for selection into a family medicine residency program that considered gender differences. This difference was not significant [42]. However, the study had a small sample size, making it difficult to detect small effects.

Because of the late introduction of group stations to our MMI, the analysed sample contained far fewer observations for group stations than for interview and simulation stations. Therefore, it was less likely to detect significant differences in group stations, and the non-significant differences in group stations need further investigation. Some findings indicate that circumstantial factors such as role expectations and gender balance within teams seem to determine the quality of leadership and collaboration [24, 43, 44]. A further investigation into whether these factors play a role in MMI group stations is certainly needed, because the results might lead to important recommendations for the structure and design of group stations.

### Language

As recommended by authors who found that non-native speakers performed worse, we encouraged interviewers, actors and raters to use clear language and avoid being judgmental towards candidates with language difficulties [20, 31]. Raters were instructed not to evaluate formal grammar or vocabulary mistakes, but to focus on the language pragmatics. Nevertheless, candidates whose first language was not German performed worse at our MMI. However, this does not imply that the MMI is biased or unfair: The MMI is a selection tool that relies heavily on communication skills in the lingua franca. Vocabulary and grammar do not have to be flawless, but recipients should comprehend the intended meaning. Thus, pragmatic language skills are key skills both in the MMI and later in clinical practice. Language proficiency is intertwined with the competencies MMIs typically aim to assess, and therefore we expected that non-native speakers would perform worse (1C). The language effect did not depend on station type, which suggests that the language problem in the MMI is general rather than context-specific. Thus, our findings do not support our assumption that simulation stations might be a better tool to assess psychosocial competencies in candidates with language difficulties. This finding might be interpreted as an indicator of bias, but the assessment reflects the demands of the clinical workplace, where German is spoken in most cases. Unfortunately, we were not able to include language in the analysis of the predictive fairness as our large sample had only a small number of non-native speakers, which posed statistical problems. The finding by Kelly et al. [31] that the MMI only predicted OSCE results within the group of EU-students could indicate problems with the predictive fairness of MMIs.

In addition, the variable *German as first language* may be a flawed operationalization for language proficiency because it does not say whether a candidate was fluent in German or not. A typical example is second-generation immigrants from Turkey who would consider Turkish as their first language because it is spoken by their parents but are also fluent in German because of their schooling. More research with more sophisticated measures of language proficiency is needed to determine the cause of non-native speakers’ difficulties in MMIs.

### Medical family background

Like Simmenroth-Nayda and Görlich [34], we did not find significant performance differences between candidates with and without a medical family background in any of the three station types (1D). Neither did we find differences in the prediction of OSCE results. This result may calm critics who suggest that interviewers and raters might be biased towards selecting the children of colleagues. Our data contains no support for the assumption that close family members might transfer important attitudes and knowledge or strategies about how to approach critical situations in a medical context. It might also be that the specific medical discipline the parent works in (e.g., surgery or psychiatry) affects whether they have had much experience with patient communication or other MMI-relevant situations. This would require more specific questions in the sociodemographic questionnaire as well as assumptions about the different medical disciplines.

Our findings strengthen the validity argument that prior medical knowledge and skills are not tested in the MMI. However, our approach of using medical family background as an indicator for prior medical knowledge and skills could be challenged. Our MMI sample did not include many candidates who had some previous training in the medical field and consequently, in this study, we could not use more direct indicators of medical knowledge.

### General limitations

This is a single-institution study and its results only apply to the Hamburg MMI. It is, however, a strength of this study that it provides data over eight cohorts and looks at basic MMI design aspects that can be of use for other institutions which use different station types.

Another limitation is the use of *z*-standardized MMI values, because this approach assumes that the cohorts themselves did not differ in their overall ability between years. It seems, however, the most adequate solution to make scores comparable between years. Other authors followed the same strategy to compare MMI values between different schools [18, 26].

### Practical implications

Performance differences between socio-demographic subgroups can be viewed from different perspectives. One would be that differences should be avoided in all circumstances. However, this view has already been dismissed as a naïve conception of test fairness [12]. From a political standpoint, the finding that a specific subgroup performs better than another could even be useful – provided the test itself is considered fair and valid (i.e., no differential test functioning, fair prediction, etc. [see 9]). For example, Henderson et al. [18] argue that the advantage for women at admission interviews could have a positive “gender-balancing effect” as men perform better at other admission criteria. In Hamburg, male candidates perform significantly better at the HAM-Nat, the natural science test preceding the MMI [38]. Between 2010 and 2017, the average percentage of female participants in the admission process was 65%, which might be influenced by the fact that women receive better school grades in Germany than men [11]. The average percentage of female candidates receiving an admission offer through the university-specific admission process (i.e., combination of candidates admitted after the HAM-Nat and candidates admitted after the MMI) was 55%. Without the MMI, this number might have been even lower. While women have slightly higher scores in our MMI simulation stations and young women exhibit the same at our interview stations, this does not fully balance out the gender effect of the HAM-Nat. If we wanted to use the MMI as a gender balancing tool, the design of our MMI would have to focus more strongly on “female” dimensions and station types that assess these dimensions (i.e., more simulations and probably interview stations, fewer group interactions). However, we should be aware that this would more strongly balance out young male candidates. Conversely, if institutions wanted to avoid gender effects, our results would suggest using more group stations. However, more knowledge is needed about different dimensions, station types and gender differences in order to derive clear suggestions.

## Conclusions

Native German speakers performed slightly better at the Hamburg MMI. The finding that young female candidates perform better at simulation and interview stations but not at group stations sheds more light on the impact of MMI design aspects on sociodemographic subgroup differences in MMIs. Nevertheless, the very small amount of variance explained does not indicate any meaningful bias. Finally, MMI performance predicted OSCE communication station performance with no differences in the prediction between sociodemographic subgroups. These results support the predictive fairness and validity of the Hamburg MMI.

## Data Availability

The datasets used and/or analysed during the current study are available from the corresponding author on reasonable request.

## Abbreviations

Abitur grade: secondary school leaving grades
HAM-Nat: Hamburg natural science test
iMed: integrierter Modellstudiengang Medizin: the name of the curriculum for medical school at the University Medical Centre Hamburg-Eppendorf
MMI: multiple mini-interview
OSCE: objective structured clinical examination
UKE: Universitätsklinikum Hamburg-Eppendorf (University Medical Centre Hamburg-Eppendorf)

## References

[1] Patterson F, Roberts C, Hanson MD, Hampe W, Eva K, Ponnamperuma G, Magzoub M, Tekian A, Cleland J (2018). 2018 Ottawa consensus statement: selection and recruitment to the healthcare professions. Med Teach..

[2] Knorr M, Hissbach J (2014). Multiple mini-interviews: same concept, different approaches. Med Educ.

[3] Rees EL, Hawarden AW, Dent G, Hays R, Bates J, Hassell AB (2016). Evidence regarding the utility of multiple mini-interview (MMI) for selection to undergraduate health programs: a BEME systematic review: BEME guide no. 37. Med Teach.

[4] Eva KW, Rosenfeld J, Reiter HI, Norman GR (2004). An admissions OSCE: the multiple mini-interview. Med Educ.

[5] Patterson F, Knight A, Dowell J, Nicholson S, Cousans F, Cleland J (2016). How effective are selection methods in medical education? A systematic review. Med Educ.

[6] Reiter H, Eva K. Vive la difference: the freedom and inherent responsibilities when designing and implementing multiple mini-Interviews. Acad Med. 2017. 10.1097/ACM.0000000000002042 29095171

[7] Knorr M, Hissbach J, Interviews HW, Patterson F, Zibarras L (2018). Multiple mini-Interviews, and selection centers. Selection and recruitment in the healthcare professions: research, theory and practice.

[8] Hissbach JC, Sehner S, Harendza S, Hampe W (2014). Cutting costs of multiple mini-interviews - changes in reliability and efficiency of the Hamburg medical school admission test between two applications. BMC Med Educ..

[9] American Educational Research Association (2014). American Psychological Association, National Council on measurement in education. Standards for educational and psychological testing.

[10] Mislevy RJ, Haertel G, Cheng BH, Ructtinger L, DeBarger A, Murray E, Rose D, Gravel J, Colker AM, Rutstein D, Vendlinski T (2013). A “conditional” sense of fairness in assessment. Educ Res Eval.

[11] Zimmermann S, Klusmann D, Hampe W (2018). Angleichung von Schulnoten für die Studierendenauswahl. Zeitschrift für Hochschulentwicklung.

[12] Wottawa H, Amelang M. Einige Probleme der "Testfairness" und ihre Implikationen für Hochschulzulassungsverfahren. Diagnostica. 1980;26(3):199–221.

[13] Knorr M, Schwibbe A, Ehrhardt M, Lackamp J, Zimmermann S, Hampe W (2018). Validity evidence for the Hamburg multiple mini-interview. BMC Med Educ..

[14] Roberts C, Walton M, Rothnie I, Crossley J, Lyon P, Kumar K, Tiller D (2008). Factors affecting the utility of the multiple mini-interview in selecting candidates for graduate-entry medical school. Med Educ.

[15] Liao SC, Hung YN, Lee CC (2018). It's a really good idea to add group interview to multiple mini-interview. Med Educ.

[16] Cameron AJ, MacKeigan LD, Mitsakakis N, Pugsley JA (2017). Multiple mini-interview predictive validity for performance on a pharmacy licensing examination. Med Educ.

[17] Ross M, Walker I, Cooke L, Raman M, Ravani P, Coderre S, McLaughlin K (2017). Are female applicants rated higher than males on the multiple mini-interview? Findings from the University of Calgary. Acad Med.

[18] Henderson MC, Kelly CJ, Griffin E, Hall TR, Jerant A, Peterson EM, Rainwater JA, Sousa FJ, Wofsy D, Franks P. Medical school applicant characteristics associated with performance in multiple mini-Interviews versus traditional Interviews: a multi-institutional study. Acad Med. 2017. 10.1097/ACM.0000000000002041 29095170

[19] Jerant A, Fancher T, Fenton JJ, Fiscella K, Sousa F, Franks P, Henderson M (2015). How medical school applicant race, ethnicity, and socioeconomic status relate to multiple mini-interview-based admissions outcomes: findings from one medical school. Acad Med.

[20] Leduc J-M, Rioux R, Gagnon R, Bourdy C, Dennis A (2017). Impact of sociodemographic characteristics of applicants in multiple mini-interviews. Med Teach..

[21] Christov-Moore L, Simpson EA, Coudé G, Grigaityte K, Iacoboni M, Ferrari PF (2014). Empathy: gender effects in brain and behavior. Neurosci Biobehav Rev.

[22] Zupan B, Neumann D, Babbage D, Willer B (2018). Sex-based differences in affective and cognitive empathy following severe traumatic brain injury. Neuropsychology..

[23] Csank PAR, Conway M (2004). Engaging in self-reflection changes self-concept clarity: on differences between women and men, and low- and high-clarity individuals. Sex Roles.

[24] Eagly AH, Karau SJ, Makhijani MG (1995). Gender and the effectiveness of leaders: a meta-analysis. Psychol Bull.

[25] Zeng XHT, Duch J, Sales-Pardo M, Moreira JAG, Radicchi F, Ribeiro HV, Woodruff TK, Amaral LAN (2016). Differences in collaboration patterns across discipline, career stage, and gender. PLoS Biol.

[26] Reiter HI, Lockyer J, Ziola B, Courneya CA, Eva K (2012). Should efforts in favor of medical student diversity be focused during admissions or farther upstream?. Acad Med.

[27] Mirwald RL, Baxter-Jones AD, Bailey DA, Beunen GP (2002). An assessment of maturity from anthropometric measurements. Med Sci Sports Exerc.

[28] Lim S, Han CE, Uhlhaas PJ, Kaiser M (2015). Preferential detachment during human brain development: age- and sex-specific structural connectivity in diffusion tensor imaging (DTI) data. Cereb Cortex.

[29] Van der Graaff J, Branje S, De Wied M, Hawk S, Van Lier P, Meeus W (2014). Perspective taking and empathic concern in adolescence: gender differences in developmental changes. Dev Psychol.

[30] Van der Graaff J, Carlo G, Crocetti E, Koot HM, Branje S (2018). Prosocial behavior in adolescence: gender differences in development and links with empathy. J Youth Adolesc.

[31] Kelly ME, Dowell J, Husbands A, Newell J, O'Flynn S, Kropmans T, Dunne FP, Murphy AW (2014). The fairness, predictive validity and acceptability of multiple mini interview in an internationally diverse student population- a mixed methods study. BMC Med Educ.

[32] Voracek M, Tran US, Fischer-Kern M, Formann AK, Springer-Kremser M (2010). Like father, like son? Familial aggregation of physicians among medical and psychology students. High Educ.

[33] Schwarzer A, Fabian F. Medizinerreport 2012: Berufsstart und Berufsverlauf von Humanmedizinerinnen und Humanmedizinern. Hannover: Hochschulinformationssystem; 2012.

[34] Simmenroth-Nayda A, Görlich Y (2015). Medical school admission test: advantages for students whose parents are medical doctors. BMC Med Educ..

[35] Hissbach JC, Klusmann D, Hampe W (2011). Dimensionality and predictive validity of the HAM-Nat, a test of natural sciences for medical school admission. BMC Med Educ..

[36] Griffin B, Harding DW, Wilson IG, Yeomans ND (2008). Does practice make perfect? The effect of coaching and retesting on selection tests used for admission to an Australian medical school. Med J Aust.

[37] Moshinsky A, Ziegler D, Gafni N (2017). Multiple mini-interviews in the age of the internet: does preparation help applicants to medical school?. Int J Test.

[38] Meyer H, Zimmermann S, Hissbach J, Klusmann D, Hampe W (2019). Selection and academic success of medical students in Hamburg, Germany. BMC Med Educ..

[39] McManus IC, Dewberry C, Nicholson S, Dowell JS (2013). The UKCAT-12 study: educational attainment, aptitude test performance, demographic and socio-economic contextual factors as predictors of first year outcome in a cross-sectional collaborative study of 12 UK medical schools. BMC Med.

[40] Aarts S, van den Akker M, Winkens B (2014). The importance of effect sizes. Eur J Gen Pract.

[41] Griffin B, Hu W (2015). The interaction of socio-economic status and gender in widening participation in medicine. Med Educ.

[42] Hofmeister M, Lockyer J, Crutcher R (2009). The multiple mini-interview for selection of international medical graduates into family medicine residency education. Med Educ.

[43] Takeda S, Homberg F (2014). The effects of gender on group work process and achievement: an analysis through self- and peer-assessment. Br Educ Res J.

[44] Gipson AN, Pfaff DL, Mendelsohn DB, Catenacci LT, Burke WW (2017). Women and leadership: selection, development, leadership style, and performance. J Appl Behav Sci.

